# Interaction between physical activity and problematic mobile phone use on suicidality in Chinese college students

**DOI:** 10.1186/s12888-020-02920-6

**Published:** 2020-10-23

**Authors:** Yang Xie, Ming Zhu, Xiaoyan Wu, Shuman Tao, Yajuan Yang, Tingting Li, Liwei Zou, Honglv Xu, Fangbiao Tao

**Affiliations:** 1 Department of Maternal, Child and Adolescent Health, School of Public Health, Anhui Medical University, No 81 Meishan Road, Hefei, 230032 Anhui China; 2 Zhongshan School of Medicine, Sun Yat-Sen University, 74 2nd Zhongshan Road, Yuexiu District, Guangzhou, 510000 Guangdong China; 3 MOE Key Laboratory of Population Health Across Life Cycle, No 81 Meishan Road, Hefei, 230032 Anhui China; 4 Anhui Provincial Key Laboratory of Population Health and Aristogenics, Anhui Medical University, No 81 Meishan Road, Hefei, 230032 Anhui China; 5 NHC Key Laboratory of Study on Abnormal Gametes and Reproductive Tract, No 81 Meishan Road, Hefei, 230032 Anhui China; 6 Department of Nephrology, The Second Hospital of Anhui Medical University, 678 Furong Road, Hefei, 230601 Anhui China; 7 School of Nursing, Anhui Medical University, 15 Feicui Road, Hefei, 230601 Anhui China

**Keywords:** Physical activity, Problematic mobile phone use, Suicidality, College students, Interaction

## Abstract

**Background:**

Previous research has found a relationship between problematic mobile phone use (PMPU) and suicidality. However, few studies have examined the interaction effects between low physical activity (PA) and PMPU on suicidality among college students. This study aimed to examine the interactions of PA and PMPU and their impact on suicidality in a school-based sample among Chinese college students.

**Methods:**

Analysis is based on date from two university in China, which recruited 4787 participants. Binomial logistic regression models were used to explore the associations of PA, PMPU with suicidal ideation and suicide attempt, as well as the interaction of PA and PMPU with suicidality.

**Results:**

The prevalence of suicide attempt and suicidal ideation were 3.5 and 7.2%, respectively. Low PA was significantly associated with suicide attempt (OR = 3.48, 95%CI: 2.52–4.81) and suicidal ideation (OR = 1.90, 95%CI: 1.46–2.46). PMPU was significantly associated with suicide attempt (OR = 3.65, 95%CI: 2.66–5.01) and suicidal ideation (OR = 2.83, 95%CI: 2.25–3.54). Interaction analysis indicated that low PA and PMPU were interactively associated with suicide attempt (OR = 9.51, 95%CI: 6.15–14.73, *P* < 0.001), RERI = 4.85(1.20–8.50), AP = 0.51(0.29–0.73), SI = 2.32(1.34–4.04). There was no additive interaction effects between PA and PMPU on suicidal ideation.

**Conclusions:**

The findings reveals that the intervention programs of suicide attempt should consider the students PA levels and PMPU.

## Background

College students are in a critical period of life, facing various of challenges and risks during the transition from school to society. Research shows that mental health issues are common among college students [1]. Suicide is the second leading cause of death among adolescents worldwide [2, 3]. A meta-analysis suggested relatively high proportions of college students have suicidal thoughts and behaviors in the past 12 months [4]. Multiple studies have shown that the incidence of 12-month suicide ideation in college students estimates in the 5% ~ 35% range, and 12-month suicide attempts range from 0.6 to 11% [5–9]. Furthermore, a meta-analysis based on Chinese college students reported that the overall pooled prevalence of suicidal ideation was 10.72% [10]. The reporting rate of Suicide attempt among college students in Chinese Chongqing was 1.7% [11]. In general, Suicidal ideation and suicide attempt are major public health problems in college students [12].

In modern society, low physical activity have been found to increase with age in adolescence [13]. Meanwhile, with the progress of economy, mobile phone becomes an essential part of life. Despite its advantages of convenience and practicability, proper use of mobile phones has become critically important that cannot be ignored. Multiple studies have shown that the rate of PMPU in adolescents is 6.3–26% [14–18]. Thus far, the interaction effects between PA and PMPU on college students’ mental health has been concerned [19]. A substantial body of research has demonstrated significant independent effects between low physical activity (LPA) and problematic mobile phone use (PMPU) on suicidality [6, 20, 21]. Yet current knowledge surrounding the relationship of PA and PMPU with suicidality is predominantly derived from Western countries [6, 20, 21].

However, there is also little research on the interaction effects between PA and PMPU on suicidal ideation and suicide attempt in college students, despite PA and PMPU being highly correlated. Therefore, the present research first sought to analyze the independent effects of PA and PMPU on suicidal ideation and suicide attempt in Chinese college students, and then sought to explore the additive interaction effects between PA and PMPU on suicidal ideation and suicide attempt.

## Methods

### Participants

The participants were recruited from a medical university and a comprehensive normal university located in Hefei, Anhui Province, and Shangrao City, Jiangxi Province, by using stratified cluster sampling. Firstly, two cities were selected by convenient sampling. Then, two schools were based on the stratified cluster sampling. Lastly, professional and class were selected randomly from medical university and comprehensive normal university. Data for this study were collected from June to July 2018. A total of 4787 college students were recruited as research objects for questionnaire surveys to assess the behaviors and mental health. Teachers and professional investigators described the questionnaires to the students and distributed them a quick response (QR) code for scanning using their cell phones in the classroom, thus allowing them to complete the electronic questionnaires. Excluding the incomplete questionnaires, there were 4624 valid questionnaires. The response rate was 96.6%. The design and data collection were reviewed and approved by the Ethics Committee of Anhui Medical University. All participants wrote informed consent for inclusion prior to the administration of the survey.

### Measures

#### Sociodemographic data

Sociodemographic data for participants were collected by questionnaire, including age, gender (male or female), grade (freshman, sophomore or junior student), registered residential background (rural or urban), only child, parents’ education level (less than high school degree or more) and self-reported family economy (bad, general or good).

#### Physical activity

The International Physical Activity Questionnaire Short Version (IPAQ-SF) was used to measure PA [22, 23]. IPAQ-SF comprised 8 items, which evaluates the frequency and duration of PA in the past week among college students. In this study, PA level was divided into low physical activity (LPA = 3.3 metabolicequivalent [METs]), moderate physical activity (MPA = 4.0 METs) and above. The criteria for low physical activity level are no physical activity reported or energy expenditure not enough to the MPA criteria. The moderate physical activity and above level is any combination of activities of three intensity ranges of at least EE ≥600 MET min/week.

#### Problematic Mobile phone use

The Self-rating Questionnaire for Adolescent Problematic Mobile Phone Use (SQAPMPU) [24] was used to measure PMPU among college students. The questionnaire comprised of 13-items that included 3 dimensions named withdrawal symptoms, craving, and physical and mental health status. Each item responded to a 5-point Likert scale (not true at all, slightly true, moderately true, strongly true, extremely true). The total scores ranged from 13 to 65, those who scores ≥29 were defined as PMPU, using the 75th percentile as the cutoff point.

#### Suicidal ideation and suicide attempt

Suicidal ideation and suicide attempt were evaluated by the 2013 Youth Risk Behavior Surveillance System in the USA [25]. There were two questions on the questionnaire that assessed suicidal ideation, all the response options were “yes” or “no”: “Have you seriously considered suicide in the last year?” and “Have you made any plans about how to commit suicide in the last year?” Meanwhile, the questions “How many suicide attempts have you actually made in the last year?” were adopted to evaluate suicide attempts, the response options were “never” or “once or more”.

### Statistical analysis

All statistical analyses were conducted using SPSS version 23.0(SPSS, Chicago, IL, USA) and an Excel spreadsheet set up by Tomas Anderson. We conducted chi-square test to compare the incidence of suicidal ideation and suicide attempt among different sociodemographic variables, PA and PMPU. Binomial logistic regression models were employed to examine the associations of PA, PMPU with suicidal ideation and suicide attempt and to evaluate the interaction of PA and PMPU with suicidality, adjusting for confounding factors. According to Table 1, not all confounding factors were significant for suicidality, thus we did not include all confounding factors in Tables 2 and 3. In this study, statistical significance was set at *P*<0.05.

**Table 1:** Frequency characteristics of suicidality in College Students (%)

Variable	*N* = 4624	Suicide attempt		χ2 value	Suicidal ideation		χ2 value
		Yes(*n* = 164) No (*n* = 4460)			Yes(*n* = 331 )No (*n* = 4293)		
Gender				1.64			0.25
Male	2058(44.5)	81(3.9)	1977(96.1)		143 (6.9)	1915 (93.1)	
Female	2566 (55.5)	83 (3.2)	2483 (96.8)		188 (7.3)	2378 (92.7)	
Grade				3.60			8.20*
Freshman	1617 (35.0)	48 (3.0)	1569 (97.0)		91 (5.6)	1526 (94.4)	
Sophomore	1619 (35.0)	57 (3.5)	1562 (96.5)		128 (7.9)	1491 (92.1)	
Junior student	1388 (30.0)	59 (4.3)	1329 (95.7)		112 (8.1)	1276 (91.9)	
Registered residence				1.84			2.41
Rural	2411 (52.1)	77 (3.2)	2334 (96.8)		159 (6.6)	2252 (93.4)	
Urban	2213 (47.9)	87 (3.9)	2126 (96.1)		172 (7.8)	2041 (92.2)	
Only child				8.41*			17.38**
Yes	1441 (31.2)	68 (4.7)	1373 (95.3)		137 (9.5)	1304 (90.5)	
No	3183 (68.8)	96 (3.0)	3087 (97.0)		194 (6.1)	2989 (93.9)	
Paternal education				1.98			0.05
< 12 years	2975 (64.3)	114 (3.8)	2861 (96.2)		211 (7.1)	2764 (92.9)	
≥ 12 years	1649 (35.7)	50 (3.0)	1599 (97.0)		120 (7.3)	1529 (92.7)	
Maternal education				0.31			2.23
< 12 years	3469 (75.0)	120 (3.5)	3349 (96.5)		237 (6.8)	3232 (93.2)	
≥ 12 years	1155 (25.0)	44 (3.8)	1111 (96.2)		94 (8.1)	1061 (91.9)	
Self-reported family economy				11.55*			34.35**
Bad	1434 (31.0)	70 (4.9)	1364 (95.1)		139 (9.7)	1295 (90.3)	
General	2903 (62.8)	83 (2.9)	2820 (97.1)		159 (5.5)	2744 (94.5)	
Good	287 (6.2)	11 (3.8)	276 (96.2)		33 (11.5)	254 (88.5)	
PA				63.73**			24.05**
Low	757 (16.4)	64 (8.5)	693 (91.5)		86 (11.4)	671 (88.6)	
Moderate and above	3867 (83.6)	100 (2.6)	3767 (97.4)		245 (6.3)	3622 (93.7)	
PMPU				72.84**			87.05**
Yes	1271 (27.5)	93 (7.3)	1178 (92.7)		164 (12.9)	1107 (87.1)	
No	33,537 (72.5)	71 (2.1)	3282 (97.9)		167 (5.0)	3186 (95.0)	

Note: * *P* < 0.05,** *P* < 0.001; Statistical methods: Chi-square test; PA is Physical activity; PMPU is problematic mobile phone use

**Table 2:** Association of PA, PMPU and suicidality in College Students

Variables	Suicide attempt		Suicidal ideation	
	Crude OR (95%CI)	Adjusted OR (95%CI)	Crude OR (95%CI)	Adjusted OR (95%CI)
PA				
Moderate and above	1.00	1.00	1.00	1.00
Low	3.48 (2.52–4.81)**	2.96 (2.13–4.13)**	1.90 (1.46–2.46) **	1.63 (1.25–2.13)**
PMPU				
No	1.00	1.00	1.00	1.00
Yes	3.65 (2.66–5.01)**	3.17 (2.30–4.38)**	2.83 (2.25–3.54)**	2.62 (2.09–3.30)**

Note: * *P* < 0.05; ** *P* < 0.001; OR is odds ratio; CI is confidence interval; PA is Physical activity; PMPU is problematic mobile phone use; Adjusted model controlled gender, grade, only child, parents’ educational level; registered residence, self-reported family economic situation

**Table 3:** Interactions of PA, PMPU and suicide attempt in College Students

Modal	PA × PMPU	β	OR(95%CI)	RERI	AP	SI
Crude	Moderate and above×No		1.00			
	Low×No	1.02	2.78 (1.66–4.63)**			
	Moderate and above×Yes	1.14	3.12 (2.10–4.65)**			
	Low×Yes	2.33	10.26 (6.65–15.82)**	5.36 (1.49–9.23)	0.52 (0.31–0.74)	2.38 (1.39–4.06)
Adjusted	Moderate and above×No		1.00			
	Low×No	0.99	2.68 (1.60–4.48)**			
	Moderate and above×Yes	1.09	2.98 (2.00–4.45)**			
	Low×Yes	2.25	9.51 (6.15–14.73)**	4.85 (1.20–8.50)	0.51 (0.29–0.73)	2.32 (1.34–4.04)

Note: * *P* < 0.05; ** *P* < 0.001; OR is odds ratio; CI is confidence interval; RERI is relative excess risk of interaction; AP is attributable proportions; SI is synergy index; PA is Physical activity; PMPU is problematic mobile phone use; Adjusted model controlled gender, grade, only child, parents’ educational level; registered residence, self-reported family economic situation

## Results

### Characteristics of participants

Table 1 displayed the frequency characteristics and group differences of the college students in current study. There were responses from 4624 college students aged between 17 and 25 years old (mean ± SD: 19.91 ± 1.27 years), 2058 were males (44.5%) and 2566 were females (55.5%). We observed low physical activity in 16.4% and PMPU in 27.5% of participants. Overall, 164 (3.5%) college students reported suicide attempt and 331 (7.2%) college students reported suicidal ideation in the last year. However, there was no sex-based significance for suicide attempt (*P* = 0.20) and suicidal ideation (*P* = 0.62). Suicide attempt and suicidal ideation revealed no statistically significant differences by registered residence and parents’ education level. College students reporting only child and bad self-reported family economy showed higher rates of having suicide attempt and suicidal ideation. Junior students showed higher rates of having suicidal ideation. Furthermore, college students who were low PA reported higher rates of having suicide attempt than have PA (8.5% VS 2.6%, respectively, P<0.001) and higher rates of having suicidal ideation (11.4% VS 6.3%,respectively, P<0.001). Higher rates of suicide attempt and suicidal ideation were also observed in those with PMPU (P<0.001, Table 1).

### Associations of PA, PMPU and suicidality

Results from binomial logistic regression analysis showed that both PA (OR = 3.48, 95%CI: 2.52–4.81) (OR = 1.90, 95%CI: 1.46–2.46) and PMPU (OR = 3.65, 95%CI: 2.66–5.01) (OR = 2.83, 95%CI: 2.25–3.54) are independently associated with suicide attempt and suicidal ideation (P<0.001 for each, Table 2). Adjusted models showed that PA (OR = 2.96, 95%CI: 2.13–4.13) (OR = 1.63, 95%CI: 1.25–2.13) and PMPU (OR = 3.17, 95%CI: 2.30–4.38) (OR = 2.62, 95%CI: 2.09–3.30) was related to suicide attempt and suicidal ideation (Table 2).

### Interactions of PA, and PMPU with suicidality

The results of a regression analysis examining the interactions of PA and PMPU with suicidality were shown in Tables 3 and 4. Table 3 shows crude and adjusted OR (95%CI) for suicide attempt in those with low PA or PMPU, have PA or PMPU, low PA or no PMPU compared with the reference group (have PA or no PMPU). There was a positive additive interaction effects between PA and PMPU on suicide attempt (*p* < 0.001), low PA college student with PMPU were more likely to be with suicide attempt (OR = 10.26, 95%CI: 6.65–15.82). After adjusting for confounding factors, the positive additive interaction effects remained significant (OR = 9.51, 95%CI: 6.15–14.73, *P* < 0.001), RERI = 4.85(1.20–8.50), AP = 0.51(0.29–0.73), SI = 2.324(1.34–4.04) (Table 3). However, there was no additive interaction effects between PA and PMPU on suicidal ideation (Table 4).

**Table 4:** Interactions of PA, PMPU and suicidal ideation in College Students

Modal	PA × PMPU	β	OR(95%CI)	RERI	AP	SI
Crude	Moderate and above×No		1.00			
	Low×No	0.85	2.34 (1.64–3.34)**			
	Moderate and above×Yes	1.16	3.19 (2.46–4.14)**			
	Low×Yes	1.36	3.90 (2.67–5.69)**	−0.63 (−2.53–1.28)	−0.16 (−0.68–0.36)	0.82 (0.46–1.48)
Adjusted	Moderate and above×No		1.00			
	Low×No	0.82	2.26 (1.58–3.24)**			
	Moderate and above×Yes	1.13	3.09 (2.38–4.02)**			
	Low×Yes	1.29	3.63 (2.48–5.31)**	−0.73 (−2.27–0.81)	−0.20 (− 0.68–0.27)	0.78 (0.46–1.34)

Note: * *P* < 0.05; ** *P* < 0.001; OR is odds ratio; CI is confidence interval; RERI is relative excess risk of interaction; AP is attributable proportions; SI is synergy index; PA is Physical activity; PMPU is problematic mobile phone use; Adjusted model controlled gender, grade, only child, parents’ educational level; registered residence, self-reported family economic situation

## Discussion

A school-based survey was conducted to investigate the independent and interaction effects between physical activity (PA) and problematic mobile phone use (PMPU) on suicide attempt and suicidal ideation in Chinese college students. The prevalence of low PA and PMPU were 16.4 and 27.5%, respectively. Participants with low PA and PMPU exhibit more suicidality. The freshman reported a lower rate of suicide attempt and suicidal ideation than sophomore and junior student, which is similar to previous study [26]. Furthermore, our data reveals that PA and PMPU have main effects on suicidality independently, as well as interaction effects between PA and PMPU on suicide attempt but not suicidal ideation.

We observed low PA in 16.4% of participants, which was similar to Brazilian adolescents (16.2%) [27]. Previous research has found an inversely relationship between PA with suicide attempt and suicidal ideation [6]. An investigation in National Youth Risk Behavior Survey in the United States suggested that increased exercise frequency was less at risk of suicide attempt and suicidal ideation [28]. However, another study in the United States showed that 13.8% of adolescents had never participated physical activity, which was lower than our study [29], and the study showed that PA was not associated with suicidality, which contradicted our study. There might be our sample was different with others. Furthermore, different evaluation criterion of PA might be influence the results of the study. The heavy learning task is a tradition in China. There are fewer physical education classes in schools when students enter the college stage, it may contribute to reduce time spent in physical activity [30].

As we know, several studies showed the existence of the relationship between PA and suicidality. A study found the level of physical activity was associated with suicide attempt among 74,186 South Korean adolescents, and the result showed gender differences [19]. In our study, there was no significance gender differences for low PA students showed higher rates of suicidality. Advanced studies are needed to confirm whether there are gender differences in the relationship between PA and suicidality.

We investigated PMPU in 27.5% of participants, which was the same as Coskun’s investigation (27.5%) [31]. Another study reported the ratio of PMPU was 21.3% in Chinese undergraduates [26], which was lower than us. This may be because the evaluation criterion for PMPU differed. PMPU was found to have an independent effect on suicide attempt and suicidal ideation in this study. Some findings have also reported that adolescents with PMPU have higher risk of suicide attempt and suicidal ideation, which is consistent with our results [21, 32]. PMPU becomes a very common health problem now [33]. Crumley et al. has found substance abuse is a risk factor for suicide attempt and suicidal ideation [34]. Roggeveen et al. has pointed out that mobile phones use may affect nervous system [35, 36]. Thus, reduce PMPU may play a significant role in the prevention of suicidality.

Plenty of studies have illuminated significant independent effects between PA and PMPU on suicide attempt and suicidal ideation. Yet study on the interaction effects of PA and PMPU on suicide attempt and suicidal ideation is lacking. Mobile phones have brought great convenience to our life, but also a series of negative influences such as exacerbating sedentary behavior [37]. A study of parent-adolescent dyads in the Minnesota found the media equipment in home environment was associated with physical activity [38]. Another study has also shown that mobile phone use is associated with low physical activity [39]. Syvaoja et al. found that PA may benefit attentional processes but mobile phone users may have adverse effect on cognitive functions [40]. Xiao Y [41] et al. reveals that the lowest frequency of physical activity participation and high electronic media use are more likely to have suicide plan. Our study demonstrates the positive additive interaction effect between low physical activity and problematic mobile phone use on suicide attempt.

## Conclusions

In our study, PA and PMPU are cross-sectional associated with suicide attempt and suicidal ideation, with interactions of PA and PMPU on suicide attempt but not suicidal ideation. Suicide prevention efforts that examine both PA and PMPU are vital for early detection of suicide attempt and suicidal ideation among college student.

## Data Availability

The datasets that were generated analyzed for the current study are not publicly available as the author does not have permission to share the data.

## Abbreviations

PA: Physical activity
LPA: Low physical activity
MPA: Moderate physical activity
PMPU: Problematic mobile phone use
OR: Odds ratio
CI: Confidence interval
QR: Quick response
EE: Energy expenditure
METs: Metabolicequivalent
IPAQ-SF: International physical activity questionnaire short version
SQAPMPU: Self-rating questionnaire for adolescent problematic mobile phone use
RERI: Relative excess risk of interaction
AP: Attributable proportions
SI: Synergy index
